# The Salford Union Infirmary

**Published:** 1909-12-25

**Authors:** F. Townson

**Affiliations:** Clerk to the Guardians. Union Offices, Salford


*To the Editor of* The Hospital.

Yours faithfully,

### THE SALFORD UNION INFIRMARY.

*To the Editor of* The Hospital.

Sir,—I am much obliged to you for inserting copy of the Guardians' Resolution of December 10th in your last issue, but find exception to your note at the foot, which is incorrect. The resolution, as forwarded to you, was carried unanimously by my Board, and not as you state. The insertion of this in your next issue will oblige.

Yours faithfully,

F. Townson,
Clerk to the Guardians.

Union Offices, Salford, December 20, 1909.

[The Report of the proceedings at the Board Meeting of the Salford Guardians on the 10th inst. in the *Manchester Evening Chronicle* states: "Ultimately the Committee's minutes, with the resolution repelling the attack, were approved by eight votes to six, three members declining to vote." Mr. Townson's statement is thus shown to be incorrect, unless it refers only to the decision to send the Board's resolution to The Hospital and to Sir Henry Burdett, which, according to the *Manchester Evening Chronicle*, was carried unanimously.—Ed. The Hospital.]

